# Attitudes Toward Older Adults: A Descriptive Cross-Sectional Study in Chilean University Students

**DOI:** 10.3390/ijerph22091450

**Published:** 2025-09-18

**Authors:** Igor Cigarroa, Jesus Alonso, María Gabriela Vallejos, Maria Antonia Parra-Rizo, Daniel Basoalto, Daniela Robles-Tapia, Yeny Concha-Cisternas, Rafael Pizarro, Juana Borja-González, Rodrigo Yañez-Yañez

**Affiliations:** 1 Escuela de Kinesiología, Facultad de Ciencias de la Salud, Universidad Católica Silva Henriquez, Santiago 7550000, Chile; 2 Departamento de Matemáticas y Estadística, División de Ciencias Básicas, Universidad del Norte, Barranquilla 080003, Colombia; jcabrera@uninorte.edu.co; 3 Aldeas Infantiles SOS, Chillán 3780000, Chile; gabriela.vallejos@aldeasinfantiles.cl; 4 Departamento de Psicología de la Salud, Facultad de Ciencias Sociosanitarias, Campus de Elche, Universidad Miguel Hernández (UMH), 03202 Elche, Spain; maria.parrar@umh.es; 5 Facultad de Ciencias de la Salud, Universidad Internacional de Valencia (VIU), 46002 Valencia, Spain; 6 Escuela de Kinesiología, Facultad de Salud, Universidad Santo Tomás, Puerto Montt 5480000, Chile; danielbasoaltoro@santotomas.cl; 7 Escuela de Nutrición y Dietética, Facultad de Salud, Universidad Santo Tomás, Santiago 8370003, Chile; danielaroblesta@santotomas.cl; 8 Escuela de Kinesiología, Facultad de Salud, Universidad Santo Tomás, Talca 3460000, Chile; yenyconchaci@santotomas.cl; 9 Universidad Arturo Prat, Iquique 1100000, Chile; 10 Facultad de Ciencias de la Rehabilitación y Calidad de Vida, Universidad San Sebastián, Sede Los Leones, Santiago 7500000, Chile; rafael.pizarro@uss.cl; 11 Departamento de Enfermería, División Ciencias de la Salud, Universidad del Norte, Barranquilla 080003, Colombia; gjuana@uninorte.edu.co; 12 CFT Santo Tomás, Punta Arenas 6200000, Chile; ryanez8@santotomas.cl

**Keywords:** ageism, aging, attitude, students, Chile

## Abstract

**Background**: Ageism is a growing concern in aging societies and can affect future professionals’ attitudes toward older adults. **Objective**: To analyze the association between attitudes toward old age, self-perceptions of aging, and contact with older adults in the community among students at a private university in Chile. **Methods**: A descriptive, cross-sectional study was conducted with 515 university students aged 18 to 42 years from 11 campuses of Santo Tomás University in Chile. Attitudes toward older adults were assessed using the Kogan’s Attitudes toward Older People Scale (KAOP), and additional data on self-perceptions of health and aging, and contact with grandparents or older adults, were collected through a self-designed questionnaire. Statistical analyses included chi-square tests, one-way ANOVA, and Bonferroni post hoc comparisons. **Results**: A majority of students (61.2%) exhibited low-level positive attitudes toward older adults. Female students and those aged 26–42 years had significantly more positive attitudes than male and younger students (*p* < 0.05). Students with a healthier self-perception of aging (*p* = 0.011) and those who maintained contact with grandparents or older adults (*p* = 0.006) showed significantly more favorable attitudes toward aging. Nationality was also associated with attitudes, with Chilean students scoring higher than their foreign counterparts. **Conclusions**: Positive attitudes toward older adults among university students were associated with gender, age, self-perception of aging, and intergenerational contact. These findings highlight the importance of integrating educational and intergenerational programs in higher education to reduce ageism and promote respectful and inclusive perceptions of aging.

## 1. Introduction

Although aging is a universal and continuous process, it manifests heterogeneously across individuals, posing significant social and policy challenges, especially in aging societies [1]. Diversity in old age represents a major challenge for countries [2,3]. Sociodemographic projections indicate that by 2050, one in four people in Latin America will be older adults [3]. In Chile, individuals over 60 years of age constitute 19.3% of the total population [4]. The InterAmerican Convention on the Protection of the Human Rights of Older Persons, which Chile ratified in 2017, defines an older person as “a person aged 60 or older, except where legislation has determined a minimum age that is lesser or greater, provided that it is not over 65 years” [5]. Meanwhile, the Chilean university system has experienced sustained growth [6].

The meanings attributed to aging and old age strongly influence how individuals perceive themselves [7]. This stereotypical construction of aging and old age is referred to as “ageism” [8]. The term “ageism” was coined in 1969 by Robert Butler. It is understood as any prejudice or discrimination for or against a particular age group [9]. Although ageism can be directed toward younger people, it is most often associated with negative attitudes and behaviors toward older adults [10]. Thus, ageism has been defined as a set of negative, socially stereotyped attitudes and prejudices held by the population against old age and the aging process [11].

In Chile, surveys have evaluated experiences of age-based discrimination among older adults. For example, the report “Ageism: Social image of old age and age discrimination,” prepared by the Aging Observatory, revealed that at least 18% of Chileans view older adults as a burden on society—making Chile one of the countries with the highest prevalence of this prejudice [12]. The “Quality of Life in Old Age” survey showed a decline in perceived unfair treatment in healthcare settings, while perceived discrimination in public services and municipalities was on the rise [3]. Common myths and stereotypes in Chile about aging and old age include the beliefs that older adults cannot learn, resist change, are inherently poor, regress to childlike behavior, and are no longer sexual beings; that old age equates to illness; and that older adults have no future [12].

Ageism is increasingly recognized as a relevant public health issue. In recent years, there has been growing interest in identifying and assessing younger people’s attitudes toward older adults [13]. The World Health Organization (WHO), within the framework of the Decade of Healthy Ageing, has identified key strategies to combat age discrimination. These include the Global Campaign to Combat Ageism, aimed at raising awareness and shifting negative perceptions of ageing; the promotion of intergenerational interventions, designed to foster interaction and cooperation between younger and older people to reduce prejudice and strengthen social cohesion; and the development of inclusive policies and legislation, ensuring equal rights and opportunities in areas such as health, employment, and community participation [14].

Globally, most studies on ageism in university populations have focused on health-related disciplines, such as nursing, medicine, or dentistry, where students are more likely to work with older adults. However, research indicates that ageist attitudes are present across all service fields [15]. Nevertheless, most of the research still focuses on health students and professionals owing to their future involvement with older adults [16]. The most frequently studied variables among university students are sociodemographic variables such as age, gender, their course of study, and level of knowledge of the aging process, which can contribute to positive attitudes toward older adults [17]. For example, a study conducted in the United States showed that women exhibited lower levels of ageist attitudes, older individuals expressed reduced levels of ageist attitudes, and students who had taken at least one of nine elective gerontology courses displayed more positive attitudes toward older adults [18]. In the case of Chile, these factors could also be decisive in understanding the phenomenon of ageism in university students.

Regarding the link between ageism and self-perception of aging in young people, the Terror Management Theory (TMT) proposed by Greenberg, et al. (1997) may offer an explanation [19]. TMT explores the role of death anxiety in shaping social behavior [1]. Encounters with older adults provoke feelings of vulnerability and act as unsettling reminders of the fragility of human life [17]. Some studies suggest that anxiety about aging and fear of death are predictors of age discrimination [18]. In the Latin American context, where aging is often associated with dependence and illness [20], such perceptions may intensify fear of one’s own aging and reinforce negative attitudes toward older adults.

In terms of contact with older adults in one’s community or grandparents, studies have shown that intergenerational contact is associated with more positive attitudes toward aging. For example, a study in Italy involving nursing students found that 69.7% of those with more positive attitudes toward old age had prior experience working with older patients [21]. A study across 25 European Union countries found that people with intergenerational friendships tended to express less ageist views. This applied to both younger and older participants [2]. In the abovementioned study, 18% of young people (ages 18–30) reported having friends aged 70 or older. Interestingly, young women were less likely than young men to have such friendships [1]. Strengthening intergenerational relationships and programs can promote active aging through cross-generational contact [22]. These interactions help improve physical and mental health, social skills, and interpersonal relationships for all involved. They are key to reducing age discrimination and social isolation among older adults, while also enhancing academic performance in young people, fostering positive perceptions of older adults, and encouraging youth participation in community activities [23]. Importantly, according to Allport’s Contact Theory (1954), sustained and meaningful contact between groups under favorable conditions can reduce prejudice; thus, intergenerational programs offer a concrete pathway to mitigating ageist stereotypes and fostering more inclusive attitudes [24].

Research shows a strong link between ageism, in the form of negative stereotypes, prejudices, and discrimination, and risks to the physical and mental health of older adults [25,26]. Exposure to negative stereotypes can lead older adults to internalize stress related to aging, adopt unhealthy behaviors, and experience depression and chronic illness, which negatively impact their behavior, cognition, emotional well-being, and overall health [27]. Conversely, having a positive attitude toward aging is associated with better self-perception of physical and mental health and higher life satisfaction [28]. Despite growing global interest in addressing ageism among young populations, there is limited evidence in Latin American contexts, particularly among students from diverse academic disciplines outside the health sciences. This study addresses this gap by examining how university students in Chile perceive aging and older adults, and what factors—such as self-perceptions of aging and intergenerational contact—may influence these attitudes.

The objective of this study aimed to examine the associations between university students’ attitudes toward aging and their self-perceptions of aging and intergenerational contact, within a sample from a private university in Chile.

## 2. Materials and Methods

Study design: The study employed a quantitative approach with a non-experimental, descriptive correlational design, and a cross-sectional temporality. Secondary data were extracted from a previous study [10]. This work stems from the research project entitled “Factors associated with attitudes toward aging among university students in Chile,” approved by the Scientific Ethics Committee of the South-Central Macrozone of Santo Tomás University, Chile (Code: 83-21). All participants provided informed consent prior to completing the questionnaires, and all procedures were conducted in accordance with the Declaration of Helsinki and the Singapore Declaration.

Sample and sampling: Students over 18 years of age enrolled at any campus and any program and year (from first to sixth) of Santo Tomás University (Chile) were invited to participate. A stratified probability sampling method was used, with strata defined by sex. The sample size of 519 was calculated using a 5% margin of error and a 95% confidence level. Four individuals were excluded for exceeding the upper age limit of 42 years, resulting in a final sample of 515 participants. The sociodemographic and educational characteristics of the sample are presented in Table 1.

Procedure: A collaboration was established with academic staff at Santo Tomás University to appoint a representative at each campus, responsible for inviting students to participate. The invitation was distributed via the university’s internal social media channels over a two-week period. These channels included a link to an online platform (Google Forms by Google Workspace) containing self-administered questionnaires used to assess attitudes toward older adults, self-perceptions of health and aging, and contact with older adults in the community. The questionnaires were available from 4 October to 8 November 2021. Participants were provided with a contact telephone number in case they had any questions or concerns.

### Variables and Evaluation Instruments

Attitudes toward older adults: The Spanish version of the Kogan’s Attitude toward Older People Scale (KAOP-S) used in this study had previously been validated in university populations [13], with a reported Cronbach’s alpha of 0.75. The KAOP-S consists of 34 statements about older adults—17 positive and 17 negative. The scale follows a six-point Likert-type format with the following response options: (A) Strongly disagree; (B) Slightly disagree; (C) Disagree; (D) Agree; (E) Slightly agree; (F) Strongly agree. The KAOP has been translated into different languages [29,30,31] and evaluated on university students from different parts of the world [32,33,34,35].

Scores range from 34 to 204, with higher total scores indicating a more positive attitude toward older adults [30]. The scale has been validated in Spanish and demonstrated good reliability (0.81), making it suitable for use in other institutions. For result interpretation, scores were categorized based on cutoff points or predefined subranges: negative attitude—34 to 118 points; low-level positive attitude—119 to 146 points, medium-level positive attitude—147 to 174 points, and high-level positive attitude—175 to 204 points [36].

Self-perception of health and aging: This variable was assessed through self-developed questions: “In general, how would you rate your health?” with the following response options: Very good/Good/Fair/Poor/Very poor, and “How do you think you will age?” with the following response options: I will age in: a very healthy way/good health/relatively good health/an unhealthy way. Current evidence suggests that self-perceived health and aging status influences attitudes towards older people [37].

Contact with older adults in the community: This variable was also assessed through self-developed questions: “Are your grandparents alive?” with the following response options: Yes, all four/Yes, three of them/Yes, two of them/Yes, one of them/I have no living grandparents, “Think of your closest grandparent(s). What is their current general health status?” with the following response options: Healthy/Healthy with a disability/Ill/Ill with mobility issues/Deceased, “Where do they live?” with the following response options: In my home/In a relative’s home/In a nursing home or other/Deceased, and “Do you have contact with your grandparents or other older adults?” with the following response options: Yes/No.

Sociodemographic data: Age; gender, with the following response options: Male/Female/Prefer not to say; nationality: “In which country were you born?” with the following response options: Chilean/Foreigner; ethnicity: “Do you belong to any ethnic group?” with the following response options: Aymara, Quechua, Atacameño, Colla, Diaguita/Mapuche, Kawésqar, Yámana, Yagán/Other/None.

Educational data: The variables and categories recorded were faculty (Social Sciences and Communications, Law, Natural Resources and Veterinary Medicine, Health, Other faculties (Sciences, Economics and Business, Education, Engineering), program (Psychology, Law, Veterinary Medicine, Occupational Therapy, Medical Technology, Nursing, Physical Therapy, Speech Therapy, Nutrition and Dietetic), other programs (Bachelor of Science, Social Work, Business Engineering, Civil Engineering), and year (first, second, third, fourth, fifth, and sixth year).

Statistical analysis: Data were analyzed using SPSS 26.0 statistical software (IBM SPSS Statistics, Chicago, IL, USA). Qualitative data were presented as frequencies and percentages, and quantitative data as mean and standard deviation. The Kolmogorov–Smirnov and Levene’s tests were used to assess the assumptions of normality and homogeneity of variance, respectively, justifying the use of parametric statistics. First, the prevalence of attitudes toward aging was described. Dependence between categorical variables was performed with the chi-square or Fisher’s exact test. One-way analysis of variance (ANOVA) was applied to compare scores on the Kogan scale (total, positive, and negative items) across sociodemographic and educational groups. When a significant effect was detected, a Bonferroni post hoc analysis was performed to identify differences between groups. The significance threshold was set at *p* < 0.05. Effect size (ES) was calculated using eta-squared (ɳ2), where ɳ2 = 0 indicates no effect, and ɳ2 = 1 indicates a perfect effect. The reference values for ɳ2 are as follows: 0.00 < ɳ2 < 0.01 = insignificant; 0.01 < ɳ2 < 0.06 = small; 0.06 < ɳ2 < 0.14 = medium; 0.14 < ɳ2 < 1.00 = large.

## 3. Results

Table 2 presents the total Kogan scale score and its categories of attitudes toward aging. The average score was 139.1 (SD = 16.6), and most of the students (61.2%) exhibited a low-level positive attitude.

Table 3 presents the association between self-perception of aging and health, indicators of contact with older adults in the community, and attitudes toward aging. A statistically significant association was observed between attitudes toward aging and both self-perception of aging (*p* = 0.011) and contact with grandparents or older adults in the community (*p* = 0.040). The hypothesis of independence was not rejected for the other variables.

Table 4 presents the Kogan scale score according to sociodemographic variables, self-perception of health and aging, and relationship with older adults in the community. A one-way ANOVA showed significant differences in attitudes toward aging as measured by the Kogan scale for the following variables: age (*p* = 0.014), gender (*p* = 0.002), nationality (*p* = 0.016), self-perception of aging (*p* = 0.011), and contact with grandparents (*p* = 0.006). A more in-depth Bonferroni post hoc analysis revealed that older university students (aged 26–42 years) had higher scores on the Kogan scale compared to other age quartiles. Additionally, women and Chilean students scored higher on the Kogan scale than men and foreign students, respectively. Students who had an unhealthy self-perception of aging and no contact with grandparents had lower scores on the Kogan scale than those with a healthier self-perception of aging and contact with grandparents, respectively.

## 4. Discussion

The main results of this study revealed that students overwhelmingly presented low-level positive attitudes toward aging. Regarding sociodemographic variables, significant differences were evident in attitudes toward aging, as measured by the Kogan scale, for variables such as age, gender, and nationality. Older university students, women, and Chileans scored higher—that is, had a more positive attitude toward aging. Furthermore, students with a healthier self-perception of aging and those who maintained contact with grandparents or older adults in their community had more favorable attitudes toward old age.

*Comparison of literature with study findings*: Regarding the Kogan scale results, this study yielded an average score of 139.1, with the main category being low-level positive attitudes (61.2% of the sample). This is consistent with a study conducted by Yañez R. et al. (2022) [10] and Pizarro-Mena R. et al. (2024) [38]. The quality of contact with older people and the positive or negative presentation of older people to others emerge as the most robust determinants of other-directed ageism [39]. The academic curriculum’s lack of training in gerontology, the limited interaction with older adults in the early years of their studies, and the predominance of negative narratives about aging may have influenced the students’ scores.

Concerning sociodemographic variables such as age, gender, and nationality, the findings agree with existing literature. The WHO World Report on Ageism (2021) stated that a recent study across 57 countries found that younger age, male sex, and lower education level were factors that increased the likelihood of ageist attitudes [1]. In the present study, older students demonstrated more positive attitudes toward aging, with those aged 26 to 42 years scoring highest on the Kogan scale. Research has shown that ageism varies by both age group and gender, with young people exhibiting higher levels of ageism [10,33,37].

Regarding gender, this study found that women had more positive attitudes toward aging than men. This is consistent with a 2021 study by López involving nursing students in Spain [17] and a 2019 study by Lira with university students in Mexico [28]. Men tended to show higher levels of ageism than women in most contemporary studies [40]. Studies on gender differences suggests that this may be because women are generally warmer, more affectionate, and empathetic, while men tend to be competitive and critical [20,40].

In terms of nationality, results indicated that Chilean students had a more positive attitude toward aging. However, the number of foreign students surveyed was small (n = 13), limiting the potential for comparative analysis. Therefore, these results should be interpreted with caution [10].

This study aligns with the theoretical framework presented in this study. There was a significant association between self-perception of aging and contact with older adults in the community: Students with a healthier self-perception of aging showed a more positive attitude toward aging. A 2020 study by Cooney et al. (2021) [18] refers to self-perception of aging as an individual’s beliefs and evaluation of the aging process. Ageism, in this context, may serve as a psychological mechanism to manage anxiety related to aging and death—by viewing older adults negatively, a person may create psychological distance. From the perspective of Terror Management Theory (TMT), ageism functions as a psychological defense mechanism against death anxiety. This theory posits that, by perceiving older adults negatively, individuals can create psychological distance that helps reduce the fear associated with their own mortality. This dynamic has been explored in research showing how fear of death influences ageist behaviors [41].

Furthermore, the present study found that students who maintained contact with grandparents and older adults in the community had more positive attitudes toward aging. This finding is consistent with a 2016 study by Ózdemir et al. (2016) on nursing students in Turkey [15], as well as with a previous study conducted in Chile, which found that university students who live or have lived with their grandparents had a better attitude toward aging compared to those who had not [38]. The WHO World Report on Ageism (2021) noted that intergenerational households—such as those in Africa, Eastern Europe, Latin America, and the Caribbean, where grandchildren live with grandparents—are common [1]. Intergenerational friendships and relationships, including grandparent–grandchild bonds, represent forms of direct intergenerational contact that may help reduce ageism [1]. One possible explanation is that such contact facilitates direct interpersonal influence, allowing younger individuals to challenge internalized stereotypes and form more nuanced perceptions of older adults through sustained and emotionally significant interactions. However, students’ self-perception of health is a more subjective and less explored area, which may present greater challenges for quantitative analysis. In literature, self-perception of health is defined as an individual’s personal or subjective assessment of their overall health status, encompassing physical, mental, and social well-being. Studies assessing general, physical, mental, and self-perceived health have found that perceived financial situation, perceived social support, age, and gender all play essential roles [42].

### Main Contributions and Clinical Implications of the Study

*Contributions*: This study provides novel, context-specific evidence on Chilean university students’ attitudes toward older adults, integrating variables such as self-perceptions of aging and intergenerational contact within their communities. Furthermore, it contributes to the growing body of literature on university students’ attitudes toward aging and older adults in Latin America.

*Clinical implications*: This study provides evidence supporting the implementation of intergenerational contact programs. There is growing support for the idea that interventions involving intergenerational contact reduce ageism against older adults [1]. These programs typically bring older and younger people together to collaborate on tasks that promote intergenerational relationships and mutual understanding [1]. There are numerous international and national examples of such contact-based interventions, including shared participation in games, gardening, art, or music therapy; mutual teaching; youth visits to long-term care facilities; collaborative service activities; in-depth interviews or discussions between older and younger people; and co-housing arrangements, also known as “shared homes” [1]. In Chile, an example is the Senior Advisors volunteer program, conducted by the Ministry of Social Development and Family. In this initiative, individuals aged 60 and above, who have retired or receive pensions, provide academic support to children in grades 1 through 8 who are part of the Families program of the Security and Opportunities Subsystem [43]. Such intergenerational activities can be implemented both at the community and the university level.

This study also explored the role of young people’s self-perception of aging and provided evidence supporting the importance of educational programs on aging targeted at university students. These programs can help improve attitudes toward aging. Existing evidence shows that greater knowledge about aging acts as a protective factor against ageism [1]. When young people acquire more knowledge in gerontology, they develop a more realistic and positive view of old age. Understanding what to expect in the future may also help them face the aging process with less anxiety [44,45]. The paradigm of the human being as a subject of rights is fundamental. This necessarily entails a life free from discrimination and the promotion of respectful treatment. Therefore, it is essential to raise awareness of ageism at all levels—in universities, schools, workplaces, the media, and civil society [44,45].

*Limitations of the study*: Like all studies, the current one has limitations. First, the findings cannot be generalized to students at other Chilean universities. This study was conducted at a single university, despite the wide diversity of both public and private universities in Chile. However, data were collected across 11 university campuses, each located in a different region of the country, thereby ensuring some degree of regional diversity. Furthermore, given the cross-sectional design of the study, causal relationships cannot be established. A longitudinal design would be necessary to determine the direction of the relationship and better understand the temporal dynamics between the variables.

Second, the recruitment method may have influenced the interpretation of the results. Most participants were enrolled in health sciences programs and were in the early years of their courses, with few final-year or engineering students. This occurred because the study coordinators at the Santo Tomás University campus were affiliated with health-related programs, making it easier to recruit students from these programs. Additionally, fifth-year students typically do not attend classes in person, as they are engaged in internships or thesis work, making it difficult for them to respond to an online survey.

Third, self-report instruments were used, which limits control over the responses. When doubts arise, participants cannot seek clarification. This was particularly relevant for the Kogan scale, which, although validated in Spanish [13], has not been validated specifically for the Chilean context. Consequently, some phrases may have been difficult to understand. In similar studies, such as one conducted in Spain, difficulty understanding the translation by Celis et al. (2012) led to a qualitative evaluation of the items through expert judgment. This process involved three experts—one in scale construction and two familiar with the construct being assessed—who made modifications to the original scale [46]. Although a researcher was available to clarify questions, it is common for students not to seek assistance and instead complete the survey independently.

Fourth, the instrument was administered online, which does not guarantee that the intended person completed the survey. However, the use of questionnaires and online data collection have proven to be an acceptable method in cross-sectional studies, especially under conditions such as the COVID-19 lockdown, which hindered in-person data collection.

## 5. Conclusions

The study found that only two factors—(i) self-perception of aging and (ii) contact with older adults in the community—were significantly linked to students’ attitudes toward aging. It emphasizes the need to continuously assess and address university students’ views on aging, regardless of their field of study, since they are future professionals who will interact with older adults. The results highlight intergenerational contact as a key strategy to prevent ageism and stress the importance of developing educational programs, workshops, and initiatives within universities to increase knowledge about aging and reduce stereotypes.

The authors recommend future longitudinal research and educational interventions to monitor and shape attitudes across the university cycle. Overall, the findings support the integration of gerontology education and intergenerational initiatives in higher education as effective tools to foster positive perceptions of aging and promote active aging, intergenerational equity, and public health, while calling on communities to build inclusive spaces free from age-based discrimination.

## Figures and Tables

**Table 1:** Sociodemographic and educational characteristics of the sample.

Variables	N (%)
Age	
*Quartile 1 (18–* *20 years)*	166 (32.2)
*Quartile 2 (* *21–23 years)*	143 (27.8)
*Quartile 3 (24–* *26 years)*	90 (17.5)
*Quartile 4 (>* *26 years)*	116 (22.5)
Gender	
*Male*	183 (35.7)
*Female*	330 (64.3)
Country of origin	
*Chilean*	502 (97.5)
*Foreigner*	13 (2.5)
Ethnicity	
*Aymara, Quechua, Atacameño, Colla, Diaguita*	45 (8.7)
*Mapuche, Kawésqar, Yámana, Yagán*	56 (10.9)
*Other ethnicity*	6 (1.2)
*People with no defined ethnicity*	408 (79.2)
Faculty	
*Social Sciences and Communications*	14 (2.7%)
*Law*	12 (2.3%)
*Natural Resources and Veterinary Medicine*	13 (2.5%)
*Health*	466 (90.5%)
*Other faculties (Sciences, Economics and Business, Education, Engineering)*	10 (1.9%)
Program	
*Psychology*	12 (2.3%)
*Law*	12 (2.3%)
*Veterinary Medicine*	13 (2.5%)
*Occupational Therapy*	63 (12.2%)
*Medical Technology*	16 (3.1%)
*Nursing*	63 (12.2%)
*Physical Therapy*	296 (57.5%)
*Speech Therapy*	11 (2.1%)
*Nutrition and Dietetics*	6 (3.1%)
*Other programs (Bachelor of Science, Social Work, Business Engineering, Civil Engineering)*	3 (2.5%)
Year	
*First year*	92 (17.9%)
*Second year*	80 (15.5%)
*Third year*	136 (26.4%)
*Fourth year*	145 (28.2%)
*Fifth and sixth year*	62 (12.0%)

Source: Prepared by the authors.

**Table 2:** Kogan scale score and categories of attitudes toward aging.

Variables	Average	SD
*Kogan score (34–204 points)*	139.1	16.6
Attitude toward aging	n	%
*Negative attitude (34–118 points)*	46	9%
*Low-level positive attitude (119–146 points)*	315	61%
*Medium-level positive attitude (147–174 points)*	144	28%
*High-level positive attitude (175–204 points)*	10	2%

Note: Qualitative data are presented as absolute and percentage frequencies, and quantitative data as mean and standard deviation (SD). n = 515. Source: Prepared by the authors.

**Table 3:** Association between self-perception of aging and health, contact with older adults in the community, and attitudes toward aging.

	Attitudes Toward Aging				Chi-Square/Fisher’s Exact
Variables	Negative	Low-Level Positive	Medium-Level Positive	High-Level Positive	*p*-Value
Self-perception of health and aging					
Self-perception of health					
*Very good*	5 (11%)	44 (14%)	21 (15%)	3 (30%)	0.164
*Good*	24 (52%)	174 (55%)	88 (61%)	6 (60%)	
*Fair*	14 (30%)	90 (29%)	35 (24%)	1 (10%)	
*Poor and very poor*	3 (7%)	7 (2%)	0 (0%)	0 (0%)	
Self-perception of aging					
*Very healthy way*	1 (2%)	17 (5%)	6 (4%)	0 (0%)	0.011
*Good health*	10 (22%)	129 (41%)	72 (50%)	4 (40%)	
*Relatively good health*	28 (61%)	138 (44%)	63 (44%)	5 (50%)	
*Unhealthy* *way*	7 (15%)	31 (10%)	3 (2%)	1 (10%)	
Contact with older adults in the community					
Are your grandparents alive?					
*Yes, all four*	8 (17%)	32 (10%)	20 (14%)	3 (30%)	0.066
*Yes, three of them*	7 (15%)	74 (24%)	33 (23%)	0 (0%)	
*Yes, two of them*	14 (31%)	81 (26%)	33 (23%)	0 (0%)	
*Yes, one of them*	14 (31%)	79 (25%)	31 (21%)	3 (30%)	
*I have no living grandparents*	3 (6%)	49 (15%)	27 (19%)	4 (40%)	
Do you have contact with your grandparents or older adults?					
*Yes*	33 (77%)	234 (88%)	109 (93%)	5 (83%)	0.040
*No*	10 (23%)	32 (12%)	8 (7%)	1 (17%)	
Think of your closest grandparent(s). What is their current general health status?					
*Healthy*	13 (30%)	93 (35%)	53 (45%)	2 (33%)	0.468
*Healthy with disabilities*	14 (33%)	100 (38%)	36 (31%)	1 (17%)	
*Ill*	11 (26%)	51 (19%)	22 (19%)	2 (33%)	
*Ill with mobility issues*	5 (11%)	22 (8%)	6 (5%)	1 (17%)	
Think of your closest grandparent(s). Where do they live?					
*At my home*	8 (18%)	53 (20%)	20 (17%)	1 (17%)	0.549
*At a relative’s home*	11 (26%)	90 (34%)	31 (27%)	1 (17%)	
*In a nursing home or other*	24 (56%)	123 (46%)	66 (56%)	4 (66%)	

Note: Qualitative data are presented as observed absolute and percentage frequencies, with expected absolute and percentage frequencies in parentheses. N = 515. Source: Prepared by the authors.

**Table 4:** Kogan score by sociodemographic variables, self-perception of health and aging, and contact with older adults in the community.

		Kogan Score	One-Way ANOVA	
Variables	n (%)	Average (SD)	*p*-Value	ES
Sociodemographic variables				
Gender				
*Male*	183 (35.7)	136.7 (15.3) **b**	0.014 *	0.012
*Female*	330 (64.3)	140.4 (17.0) **a**		
Age				
*Quartile 1 (18–21 years)*	166 (32.2)	136.6 (16.4) **b**	0.001 *	0.032
*Quartile 2 (22–23 years)*	143 (27.8)	138.4 (16.8) **b**		
*Quartile 3 (24–25 years)*	90 (17.5)	137.8 (14.7) **b**		
*Quartile 4 (26–42 years)*	116 (22.5)	144.5 (16.9) **a**		
Country of origin				
*Chilean*	502 (97.5)	139.4 (16.6) **a**	0.016 *	0.011
*Foreigner*	13 (2.5)	128.2 (9.9) **b**		
Ethnicity				
*Aymara, Quechua, Atacameño, Colla, Diaguita*	45 (8.7)	137.7 (17.4)	0.479	0.005
*Mapuche, Kawésqar, Yámana, Yagán*	56 (10.9)	139.8 (15.1)		
*Other*	6 (1.2)	129.5 (6.7)		
*None*	408 (79.2)	139.3 (16.7)		
Self-perception of health and aging				
Self-perception of health				
*Very good*	73 (14.2)	141.2 (17.5)	0.185	0.009
*Good*	292 (56.7)	139.3 (16.7)		
*Fair*	140 (27.2)	138.2 (15.9)		
*Poor*	10 (1.9)	129.9 (13.5)		
Self-perception of aging				
*Very healthy*	24 (4.7)	138.5 (14.7) **ab**	0.011 *	0.021
*Healthy*	215 (41.7)	141.3 (16.3) **a**		
*Partially healthy*	234 (45.4)	138.3 (16.8) **ab**		
*Unhealthy*	42 (8.2)	132.5 (16.3) **b**		
Contact with older adults in the community				
Are your grandparents alive?				
*Yes, all four*	63 (12.2)	141.3 (17.6)	0.068	0.017
*Yes, three of them*	114 (22.1)	138.4 (15.6)		
*Yes, two of them*	128 (24.9)	136.7 (16.0)		
*Yes, one of them*	127 (24.7)	138.6 (16.3)		
*I have no living grandparents*	83 (16.1)	143.0 (17.8)		
Do you have contact with your grandparents or older adults?				
*Yes*	381 (74.0)	139.1 (15.8) **a**	0.006 *	0.018
*No*	51 (9.9)	132.5 (18.3) **b**		
Think of your closest grandparent(s). What is their current general health status?				
*Healthy*	161 (31.3)	140.7 (15.6)	0.160	0.012
*Healthy with disabilities*	151 (29.3)	137.1 (16.3)		
*Ill*	86 (16.7)	136.8 (17.3)		
*Ill with mobility issues*	34 (6.6)	137.0 (15.5)		
Think of your closest grandparent(s). Where do they live?				
*At my home*	82 (15.9)	136.3 (16.7)	0.411	0.004
*At a relative’s home*	133 (25.8)	138.4 (14.0)		
*In a nursing home or other*	217 (42.1)	139.1 (17.3)		

Note: Qualitative data are presented as absolute frequency and percentage, and quantitative data as mean and standard deviation (SD). N = 515. * *p* < 0.05. A Bonferroni post hoc analysis was performed to identify differences between groups. **a**, **b** Different letters in the same column indicate significant differences between groups. Source: Prepared by the authors.

## Data Availability

The data presented in this study are available on request from the corresponding author due to privacy and legal reasons.
